# Supplementary material for: Improved bounds on Lorentz violation from composite pulse Ramsey spectroscopy in a trapped ion
Source: Nat Commun. 2022 Nov 27;13:7314. doi: 10.1038/s41467-022-34818-0 (PMC9701673; doi:10.1038/s41467-022-34818-0)
Supplement: Supplementary file 1 — Supplementary Information [file 41467_2022_34818_MOESM1_ESM.pdf]

Supplementary materials to: Improved bounds on Lorentz violation from composite pulse Ramsey spectroscopy in a trapped ion

Laura S. Dreissen[1]*, Chih-Han Yeh[1], Henning A. Fürst[1,2], Kai C. Grensemann[1], Tanja E. Mehlstäubler[1,2]

[1]Physikalisch-Technische Bundesanstalt, Bundesallee 100, 38116, Braunschweig, Germany
[2]Institut für Quantenoptik, Leibniz Universität Hannover, Welfengarten 1, Hanover, Germany

Supplementary Note 1

The elements of the $c_{\mu\nu}$ tensors are dependent of the local laboratory frame. In order to compare our results with those from other experiments, we transform the components of the local c_{mn} tensor to the sun-centered, celestial-equatorial frame (SCCEF). We follow a similar derivation as given in ref. 1. The relation between the components of c_{mn} in the lab frame and c_{MN} in the SSCEF is given by

$$c_{mn} = \Lambda_m^M \Lambda_n^N c_{MN} \,, \tag{1}$$

where Λ is the Lorentz transformation matrix, which consists of rotations and boosts in the lab frame relative to the SCCEF. Our lab frame has its origin at a colatitude of $\chi = 37.7°$ and a longitude of $\lambda = 10.5°$ (PTB Braunschweig, Germany). In the local coordinate system, $\hat{x}$ points towards the east, $\hat{y}$ points towards the north and $\hat{z}$ points upward. The rotation matrix that maps the SCCEF coordinate frame to that of the lab is given by

$$\mathbf{R} = \begin{pmatrix} -\sin(\omega_\oplus T) & \cos(\omega_\oplus T) & 0 \\ -\cos(\chi)\cos(\omega_\oplus T) & -\cos(\chi)\sin(\omega_\oplus T) & \sin(\chi) \\ \sin(\chi)\cos(\omega_\oplus T) & \sin(\chi)\cos(\omega_\oplus T) & \cos(\chi) \end{pmatrix} \,, \tag{2}$$

where $\omega_\oplus/2\pi = 1/23.934\,\mathrm{h}$ is the angular frequency of a sidereal day. Our quantization magnetic field B lies in the xy plane and points 20° south of east. Similar as in ref. 1, we calculate a virtual location on Earth's surface, where B points vertically upward by only changing the origin of the coordinate system, not affecting transformation formulas between the lab frame and the SCCEF frame. This yields $\chi_n = 102.1°$ and $\lambda_n = 84.4°$. Substituting $\chi = \chi_n$ and $\lambda = \lambda_n$ values in equation (2) and setting $T = 0$ at the moment the z axis of the new location points towards the Sun on the day of the Vernal equinox, in this case 03:59:24 UTC, March 20, 2021 yields the proper transformation equations.
The boost of the experimental frame as seen from the SCCEF is given by

$$\boldsymbol{\beta} = \begin{pmatrix} \beta\sin(\Omega_\odot T) \\ -\beta\cos(\eta)\cos(\Omega_\odot T) \\ -\beta\sin(\eta)\cos(\Omega_\odot T) \end{pmatrix} \,, \tag{3}$$

where $\Omega_\odot/2\pi = 1/(365.256 \times 24\mathrm{h})$ is the angular frequency of a sidereal year, $\beta = 1 \times 10^{-4}$ is the magnitude of the boost from the orbital velocity and $\eta = 23.4°$ is the angle between the ecliptic plane and the Earth's equatorial plane. Here the boost from the Earth's rotation has been neglected as it is two orders of magnitude smaller ($\beta_L = 1.5 \times 10^{-6}$). Using the rotations and boosts, the Lorentz

*Corresponding author. Email: laura.dreissen@ptb.de

ω_j	C_j	S_j
$\omega_\oplus$	$-3\sin(2\chi)c_{XZ} + 2c_{TY}\beta_L$	$-3\sin(2\chi)c_{YZ} - 2c_{TX}\beta_L$
$2\omega_\oplus$	$-\frac{3}{2}(c_{XX} - c_{YY})\sin^2(\chi)$	$-3c_{XY}\sin^2(\chi)$
$\Omega_\odot$	$-\frac{1}{2}\beta(3\cos(2\chi)+1)(c_{TY}\cos(\eta) - 2c_{TZ}\sin(\eta))$	$\frac{1}{2}\beta c_{TX}(3\cos(2\chi)+1)$
$2\Omega_\odot$	0	0
$\Omega_\odot - \omega_\oplus$	$\frac{3}{2}\beta c_{TX}\sin(\eta)\sin(2\chi)$	$-\frac{3}{2}\beta\sin(2\chi)[c_{TY}\sin(\eta) + c_{TZ}(1+\cos(\eta))]$
$\Omega_\odot + \omega_\oplus$	$\frac{3}{2}\beta c_{TX}\sin(\eta)\sin(2\chi)$	$-\frac{3}{2}\beta\sin(2\chi)[c_{TZ}(1-\cos(\eta)) - c_{TY}\sin(\eta)]$
$2\Omega_\odot - \omega_\oplus$	0	0
$2\Omega_\odot + \omega_\oplus$	0	0
$\Omega_\odot - 2\omega_\oplus$	$-3\beta c_{TY}\cos^2(\eta/2)\sin^2(\chi)$	$-3\beta c_{TX}\cos^2(\eta/2)\sin^2(\chi)$
$\Omega_\odot + 2\omega_\oplus$	$3\beta c_{TY}\sin^2(\eta/2)\sin^2(\chi)$	$-3\beta c_{TX}\sin^2(\eta/2)\sin^2(\chi)$
$2\Omega_\odot - 2\omega_\oplus$	0	0
$2\Omega_\odot + 2\omega_\oplus$	0	0

Table S1 Contributions of c_{MN} to $C_0^{(2)}$. The angular frequencies and the corresponding amplitudes contributing to $C_0^{(2)}$ in the SCCEF as a function of the colatitude χ and the angle between the ecliptic plane and the Earth's equatorial plane η.

transformation that maps $c_{\mu\nu}$ from the SCCEF to the lab frame is given by

$$\Lambda = \begin{pmatrix} 1 & -\beta^1 & -\beta^2 & -\beta^3 \\ -(R\cdot\beta)^1 & R^{11} & R^{12} & R^{13} \\ -(R\cdot\beta)^2 & R^{21} & R^{22} & R^{23} \\ -(R\cdot\beta)^3 & R^{31} & R^{32} & R^{33} \end{pmatrix}, \tag{4}$$

Using equation (4), the parameter $C_0^{(2)}$ can now be expressed in terms of components of c_{MN} in the SCCEF using the Lorentz transformation. It is given by

$$C_0^{(2)} = A_0 + \sum_j [C_j\cos(\omega_j T) + S_j\sin(\omega_j T)], \tag{5}$$

where A_0 is a constant offset, ω_j contains all linear combinations of $\omega_\oplus$ and $\Omega_\odot$, and C_j and S_j are the respective amplitudes as given in Tab. S1.
With the high-pass filter applied to the data, we are sensitive only to signals that oscillate at frequencies larger than $\nu_c = 5$ μHz. Therefore the Lorentz violating signal is given by

$$\begin{aligned} C_0^{(2)} &= -3\sin(2\chi)c_{XZ}\cos(\omega_\oplus T) - 3\sin(2\chi)c_{YZ}\sin(\omega_\oplus T) \\ &- \frac{3}{2}(c_{XX} - c_{YY})\sin^2(\chi)\cos(2\omega_\oplus T) - 3c_{XY}\sin^2(\chi)\sin(2\omega_\oplus T). \end{aligned} \tag{6}$$

Combining equation (6) with the sensitivity of $\kappa_{\rm LV}$ to $C_0^{(2)\,2}$ yields

$$\begin{aligned} \kappa_{LV} &= 2\pi \times 5.1 \times 10^{15} \times [-3\sin(2\chi)c_{XZ}\cos(\omega_\oplus T) - 3\sin(2\chi)c_{YZ}\sin(\omega_\oplus T) \\ &- \frac{3}{2}(c_{XX} - c_{YY})\sin^2(\chi)\cos(2\omega_\oplus T) - 3c_{XY}\sin^2(\chi)\sin(2\omega_\oplus T)]\,. \end{aligned} \tag{7}$$

The stability of $\kappa_{\rm LV}$ was measured to be $\sigma_\kappa = 372(9)\,\mathrm{mrad\,s^{-1}}\tau^{-1/2}$, from which we can extract the stability of $C_0^{(2)}$ to be $\sigma_{C_0^{(2)}} = 1.16 \times 10^{-17}\,\tau^{-1/2}$.

Supplementary Note 2

The quadratic term in the free Hamiltonian for state $|J, m_j\rangle$ interacting with a magnetic field $\mathbf{B} = B_z\hat{z}$ is given by $\mathcal{H}_{\rm quad} = \kappa J_z^2$. It scales with $\kappa = \kappa_{\rm q} + \kappa_{\rm LV}$, where the first term is given by the quadrupole shift and the second term is given by a potential LV. The quadrupole shift can be calculated using [3]

$$\Delta\nu_{\rm quad} = \frac{1}{4}\frac{J(J+1) - 3m_j^2}{J(2J-1)}\frac{1}{h}\Theta\left({}^2F_{7/2}\right)\frac{\mathrm{d}E}{\mathrm{d}z}[3\cos^2(\beta) - 1]\,, \tag{8}$$

The quadrupole moment of the $^2F_{7/2}$ state is given by $\Theta\left(^2F_{7/2}\right) = -0.0297(5)ea_0^2{}^4$, where e is the electron charge and a_0 is the Bohr radius. The electric field gradient is given by $\mathrm{d}E/\mathrm{d}z = -m_{\mathrm{ion}}\omega_z^2/q$ for a single trapped ion with mass m_{ion} and charge q at the equilibrium position of the trap. The angle

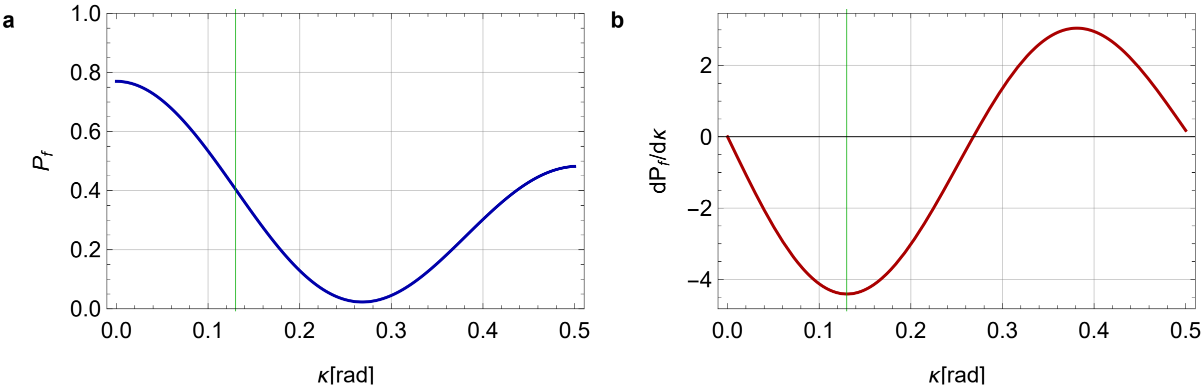

Fig. S1 The sensitivity of the measured quantity P_{f} to variations in κ. **a** The calculated final population P_{f} as a function of κ at $T = 1.15$ s, where the experimentally achieved contrast of 0.77(6) was taken into account. **b** The derivative $\mathrm{d}P_{\mathrm{f}}/\mathrm{d}\kappa$ as a function of κ at $T = 1.15$ s. The measurement sensitivity at $\kappa_{\mathrm{q}} = 0.13$ (green vertical line in **a** and **b**) is given by $\mathrm{d}P_{\mathrm{f}}/\mathrm{d}\kappa|_{\kappa=0.13} = -4.4$.

between the quantization axis and the principle axis of the trap is in our case $\beta = 25°$. The corresponding value for κ_{q} is given by

$$\frac{\kappa_{\mathrm{q}}}{2\pi} = -\frac{3}{4J(2J-1)}\frac{1}{h}\Theta\left(^2F_{7/2}\right)\frac{\mathrm{d}E}{\mathrm{d}z}\left[3\cos^2(\beta)-1\right]. \tag{9}$$

At typical values of the axial secular frequency in our trap $\omega_{\mathrm{ax}}/2\pi = (200-290)\,\mathrm{kHz}$, the quadrupole shift is $\nu_{\mathrm{quad}} = 60-125\,\mathrm{mHz}$, corresponding to $\kappa_{\mathrm{q}} = 0.075-0.150\,\mathrm{rad/s}$.
The fraction P_{f} retrieved back into the $m_j = \pm 1/2$ state at the end of the rf sequence is dependent on the acquired phase κT_{D}, where T_{D} is the Ramsey dark time. At $\kappa T_{\mathrm{D}} = 0.15$ rad, the slope $|\mathrm{d}P_{\mathrm{f}}/\mathrm{d}\kappa|$ is maximum and the highest measurement sensitivity is reached. In the experiment, $\kappa = 0.13(3)$ for which an optimum Ramsey dark-time of $T_{\mathrm{D}} = 1.15\,\mathrm{s}$ is found. Using the average achieved contrast of $(P_{\mathrm{f,max}} - P_{\mathrm{f,min}})/(P_{\mathrm{f,max}} + P_{\mathrm{f,min}}) = 0.77$, P_{f} and $\mathrm{d}P_{\mathrm{f}}/\mathrm{d}\kappa$ are calculated as a function of κ, as shown in Fig. S1 a and b, respectively. At $\kappa_{\mathrm{q}} = 0.13(3)$, the sensitivity of P_{f} to variations of κ is calculated to be $\mathrm{d}P_{\mathrm{f}}/\mathrm{d}\kappa|_{\kappa=0.13(3)} = -4.4(4)$. The uncertainty on κ_{LV} stemming from $\Delta(\mathrm{d}P_{\mathrm{f}}/\mathrm{d}\kappa|_{\kappa=0.13(3)}) = 0.4$ is added to $\Delta\kappa_{\mathrm{LV}}$ in quadrature.

Supplementary Note 3

The E3 servo sequence is based on four measured populations at half the linewidth of the two opposite Zeeman transitions $\left|^2S_{1/2}, m_j = \pm 1/2\right\rangle \rightarrow \left|^2F_{7/2}, m_j = \pm 1/2\right\rangle$. From the average value of the four measured data points the excitation probability of the E3 transition (p_{E3}) can be calculated. The E3 servo sequence is repeated every 50 data points throughout the measurement campaign, allowing us to monitor p_{E3} during this time.
Slow drifts of p_{E3} are observed on timescales of $\tau < 2.5\,\mathrm{days}$, corresponding to Fourier frequencies of $\omega/2\pi < 5\,\mu\mathrm{Hz}$, due to changes in, e.g., beam pointing and ambient noise. The quantity P_{f} is detected via de-excition from the $^2F_{7/2}$ on the E3 transition and it is, therefore, highly correlated with p_{E3}. To quantify the correlation, data points are averaged over a time span of about one day and Pearson's correlation factor is calculated to be 0.9. The number of measurements per averaged data point are not equal for p_{E3} and P_{f}. Therefore, the standard deviation of the two data sets are significantly different. For visualization purposes, the measured quantities are scaled by their respective standard deviation and plotted together with the 95% confidence interval, see Fig. S2. Note that Pearson's correlation factor differs from 1, because the p_{E3} and P_{f} are not measured at exactly the same time, but rather in an alternating fashion.
The measured data points P_{f} are corrected for slow drifts of p_{E3}. Residual slow variations that are not clearly connected to p_{E3} are observed in the data at $\omega/2\pi = 1.65\,\mu\mathrm{Hz}$, related to a fluctuation on the

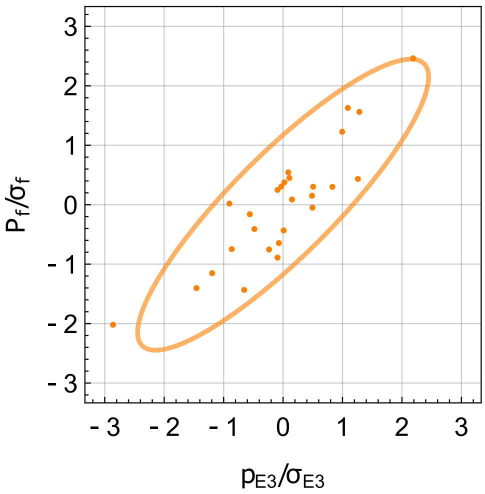

Fig. S2 Correlation plot. The correlation between the E3 excitation probability p_{E3} and the measured quantity P_{f} averaged over a time span of about one day is plotted. The data points show $p_{\mathrm{E3}}/\sigma_{\mathrm{E3}}$ and $P_{\mathrm{f}}/\sigma_{\mathrm{f}}$ after subtracting the offset of 22.6 and 9.2, respectively. Pearson's correlation is calculated to be 0.90. The ellipse indicates the 95% confidence interval.

timescale of a week. This Fourier component is removed from the data with a high-pass filter using a Hamming window with a cut-off frequency of $\nu_c = 5\,\mu$Hz. Bounds on LV at frequencies $\omega_\oplus$ and $2\omega_\oplus$ are extracted from the filtered data using a fit to equation (7).
To investigate the possible influence from the filter on the result, data points are simulated with Fourier components at $\omega/2\pi = 1.5\,\mu$Hz, $\omega = \omega_\oplus$ and $\omega = 2\omega_\oplus$, mimicking the observed slow drifts and a hypothetical LV signal, respectively. Exactly the same time stamps are used for the simulated data as for the experiment. The high-pass filter is applied to the simulated data with different values of ν_c, after which it is fitted to extract the amplitudes at $\omega_\oplus$ and $2\omega_\oplus$. The retrieved amplitudes do not significantly differ from the simulated amplitudes for cut-off frequencies between $2 < \nu_c < 10\,\mu$Hz. To validate this, the actual experimental data is also filtered with different values of ν_c in the range of $2 < \nu_c < 10\,\mu$Hz. The extracted amplitudes at $\omega_\oplus$ and $2\omega_\oplus$ from the fit to equation (7) do not show a significant deviation for different values of ν_c in this range.

References

1. Sanner, C. *et al.* Optical clock comparison for Lorentz symmetry testing. *Nature* **567** (7747), 204–208 (2019) .

2. Shaniv, R. *et al.* New methods for testing Lorentz invariance with atomic systems. *Phys. Rev. Lett.* **120**, 103202 (2018) .

3. Roos, C. F., Chwalla, M., Kim, K., Riebe, M. & Blatt, R. Precision spectroscopy with entangled states: Measurement of electric quadrupole moments. *AIP Conference Proceedings* **869** (1), 111–118 (2006) .

4. Lange, R. *et al.* Coherent suppression of tensor frequency shifts through magnetic field rotation. *Phys. Rev. Lett.* **125**, 143201 (2020) .
